# Improved Human Age Prediction by Using Gene Expression Profiles From Multiple Tissues

**DOI:** 10.3389/fgene.2020.01025

**Published:** 2020-09-24

**Authors:** Fayou Wang, Jialiang Yang, Huixin Lin, Qian Li, Zixuan Ye, Qingqing Lu, Luonan Chen, Zhidong Tu, Geng Tian

**Affiliations:** ^1^School of Computer and Data Engineering, Ningbo Institute of Technology, Zhejiang University, Ningbo, China; ^2^Key Laboratory of Systems Biology, Center for Excellence in Molecular Cell Science, Innovation Center for Cell Signaling Network, Institute of Biochemistry and Cell Biology, Shanghai Institute of Life Sciences, Chinese Academy of Sciences, Shanghai, China; ^3^Department of Genetics and Genomic Sciences, Icahn School of Medicine at Mount Sinai, New York, NY, United States; ^4^Geneis Beijing Co., Ltd., Beijing, China; ^5^Qingdao Geneis Institute of Big Data Mining and Precision Medicine, Qingdao, China; ^6^Reproductive Center, Northwest Women and Children's Hospital, Xi'an, China

**Keywords:** age prediction, aging, gene expression, RNA sequencing, genotype-tissue expression (GTEx)

## Abstract

Studying transcriptome chronological change from tissues across the whole body can provide valuable information for understanding aging and longevity. Although there has been research on the effect of single-tissue transcriptomes on human aging or aging in mice across multiple tissues, the study of human body-wide multi-tissue transcriptomes on aging is not yet available. In this study, we propose a quantitative model to predict human age by using gene expression data from 46 tissues generated by the Genotype-Tissue Expression (GTEx) project. Specifically, the biological age of a person is first predicted via the gene expression profile of a single tissue. Then, we combine the gene expression profiles from two tissues and compare the predictive accuracy between single and two tissues. The best performance as measured by the root-mean-square error is 3.92 years for single tissue (pituitary), which deceased to 3.6 years when we combined two tissues (pituitary and muscle) together. Different tissues have different potential in predicting chronological age. The prediction accuracy is improved by combining multiple tissues, supporting that aging is a systemic process involving multiple tissues across the human body.

## Introduction

Different people may age at different rates as revealed by recent studies (Li et al., 2009; Horvath, 2013). Some people appear younger than their chronological age, and others appear older. In an extreme case, a 16-year-old girl without any known genetic syndromes or chromosomal abnormalities appeared to stop growing and looked like an infant (Walker et al., 2009). It is a challenge to identify her “actual” age. Many factors, for instance, lifestyle, and environmental factors, can hasten or delay aging (Feldman et al., 1994; Hultsch et al., 1999). Thus, a set of biomarkers that can reliably reflect real age has practical value. There are special cases in which such age biomarkers are particularly useful. For example, people may need to verify an athlete's age in sporting events such as the Olympic Games or to determine a suspect's age in certain forensic cases.

Different types of biomarkers have been proposed to quantify human age (Li et al., 2009). Physical parameters, such as visual acuity, auditory threshold, and maximum work rate, have been used as indicators of aging for more than three decades (Furukawa et al., 1975; Borkan and Norris, 1980). Other criteria, such as gray hair and skin wrinkles, can also reflect chronological age (Van Neste and Tobin, 2004). However, these parameters often do not provide accurate estimation of age and cannot reveal the internal molecular changes of the human body or the underlying aging mechanisms.

With the rapid development of high-throughput technologies, genomic, and epigenetic data are accumulating to an unprecedented status. This provides a new route of estimating aging at the molecular level. Associations between epigenetic variations (e.g., DNA methylation and histone modification) and age have been reported (Fraga and Esteller, 2007). It is manifested that gene expression and the methylation profile of blood (Bocklandt et al., 2011; Hannum et al., 2013; Horvath, 2013), the gene expression profile of brain (Fraser et al., 2005), and telomere length (Harley et al., 1990; Benetos et al., 2001) are good indicators of age in human and other primates. In addition, these biomarkers may also provide candidate targets for intervention to extend the human life span (Baker and Sprott, 1988).

Previous studies on age prediction using gene expression mainly rely on single tissues, such as blood or brain. The predictive ability of different tissues had not been thoroughly studied. Because aging is a concordant process involving multiple tissues (Kujoth et al., 2005), it might be effective to build an age-prediction model with information from multiple tissues. In this study, we built an optimal age prediction model by using the Genotype-Tissue Expression (GTEx) profile among 46 human tissues and then compared the predictive efficiency of a single tissue and combining two tissues.

## Methods

### Tissue Gene Expression and Data Preprocessing

From the GTEx (V6), the gene expression profiles from 46 tissues were used. A detailed description of sample collection, RNA preparation, RNA sequencing, gene expression estimation, etc., are listed in the GTEx consortium paper (The GTEx Consortium, 2015). We first normalized the original gene expression data from GTEx via quantile normalization.

### Pearson Correlation for Selecting Age-Associated Genes

The genes in each tissue were ranked based on the Pearson correlation of donor age and corresponding gene expression. Then, we picked top genes from 50 to 6400 with multiples of 2 as a model input and tuned it by 10-fold cross-validation (CV).

### Accuracy of the Models

In this paper, we use root-mean-square error (RMSE) to measure the accuracy of the models. RMSE is a frequently used measure of the differences between values (sample or population values) predicted by a model or between an estimator and the values observed. In the age-prediction models, we use RMSE to measure the quality of the model: the smaller the RMSE, the higher the accuracy of the model—and on the contrary, the lower the accuracy of the model. The RMSE of predicted value ŷ, a regression's dependent variable *y*, is computed for different predictions as the square root of the mean of the squares of the deviations:

$$\begin{array}{l} \text{RMSE}=\sqrt{\frac{\sum_{i=1}^{n}{\left(y_{i}-{ŷ}_{i}\right)}^{2}}{n}}. \end{array}$$

### Prediction Based on Single Tissue

Our age-prediction model is based on the elastic net algorithm (Zhou and Hastile, 2005). The elastic net algorithm has a sparsity property and favors grouping effects so that strongly correlated predictors tend to be in or out of the model together. These properties let the method specifically fit our study because gene expression is highly interrelated, and our prediction model relies on only a small number of genes. The age-prediction process is formulated as follows:

$$\begin{array}{l} \hat{\omega }=\arg\underset{\omega }{\underset{︸}{\min}}\{\sum_{i=1}^{M}{\left(Age_{i}-\omega_{0}-\sum_{j=1}^{N}x_{ij}\omega_{j}\right)}^{2}\\ +\lambda (\alpha \sum_{j=1}^{N}|\omega_{j}|+\frac{1-\alpha }{2}\sum_{j=1}^{N}\omega_{j}^{2})\;\}, \end{array}$$

where *Age*_*i*_ is the chronological age of the donor of sample *i* with 1 ≤ *i* ≤ *M, M* is the number of samples in a particular tissue, *x*_*ij*_ is the log2-transformed expression of gene *j* with 1 ≤ *j* ≤ *N* for sample *i*, *N* is the number of preselected genes in the tissue, ω_0_ is the intercept, ω_*j*_ is the weight of gene *j*, $\hat{\omega }$ is the predicted value of ω, 0 ≤ α ≤ 1 is a parameter to balance the *L*_1_ (e.g., lasso) and *L*_2_ (e.g., ridge regression) penalty, and λ is the lasso parameter. The two parameters α and λ are optimized by a 10-fold CV. After ω_0_ and ω_*j*_ (1 ≤ *j* ≤ *N*) are determined, the following equation is used to predict age for a new sample *y* with an expression level known for selected genes:

$$\begin{array}{l} \text{Age}=\omega_{0}+\sum_{j=1}^{N}y_{j}\omega_{j}. \end{array}$$

It is worth noting that the main purpose of this study is to compare the predictive capability of a single tissue with double tissues. Because the main focus is not to identify the “best” predictive models, we do not compare the performance of elastic net with other machine learning methods. However, given the wide application of elastic net in age prediction (Hannum et al., 2013), we consider it to be an appropriate choice to serve the main purpose of this work.

### Parameter Tuning and Model Selection

To identify the best age-prediction model, we applied the 10-fold CV strategy to the analysis. In addition, we bootstrapped the CV process 100 times and averaged the validation RMSE and Pearson correlation coefficient (PCC) to reduce the potential bias that originated from random sampling when splitting the sample into training and testing sets.

As stated above, there are three model parameters, namely the preselection threshold N, parameter α to balance the lasso and ridge regression penalties, and lasso parameter λ. These parameters are tuned by 10-fold CV. Specifically, we let *N* increase from 50 to 6400 by multiples of 2, α increase from 0 to 1 with a step-wise addition of 0.01, and λ increase from 2^−10^ to 2^10^ with multiples of 2. The set of parameters yielding the lowest averaged validation RMSE in the 100 bootstrapped, 10-fold, CV runs were chosen as the optimal parameters for single and double tissue. It is of note that we reranked and selected genes (based on the 9 fold training data) in each CV to avoid overfitting.

### Prediction Using Gene Expression Data of Two Tissues

Because the number of overlapping samples among three tissues are often less than 70, we only analyzed samples that came from two tissues. To balance the contribution of individual tissue, an equal number of top gene expression profiles from each tissue were combined as features in the prediction model. A similar analysis was then applied to tune the model parameters. The performance of each tissue and double tissues were evaluated by RMSE from both validation and testing data.

### DAVID Analysis

The DAVID (6.7) (Huang et al., 2009) (https://david.ncifcrf.gov/tools.jsp) bioinformatics resource consists of an integrated biological knowledge base and analytic tools aimed at systematically extracting biological meaning from large gene/protein lists. We can use DAVID, a high-throughput and integrated data-mining environment, to analyze gene functional classification, functional annotation charts, or clustering and functional annotation tables through gene lists derived from our age-prediction models. By following this protocol, investigators are able to gain an in-depth understanding of the aging themes in lists of genes that are enriched in genome-scale studies.

## Results

### Using GTEx Gene Expression Profile as Data Input

We develop a computational framework to predict donor age depending on the gene expression profile of one single or two tissues generated from GTEx (Version 6). GTEx contains expression profiles of more than 41,298 genes in 46 human tissues. There are 34,443 genes and 8,375 samples that passed the quality control and data processing procedure that was used as the benchmark data in this study. Detailed information on the samples for 46 tissues is provided in Table 1. As can be seen from Table 1, the ages of donors range from 20 to 70, and the number of samples varies from 71 to 430 for each tissue.

**Table 1:** Sample Information of 46 tissues in GTEX.

**Tissue**	**Number**	**Minimum**	**Maximum**	**Median**	**Mean**	**NumMen**	**NumWomen**	**Proportion**
Adipose_subcutaneous	350	21	70	55	52	219	131	1.672
Adipose_visceral_(omentum)	227	21	70	54	52	145	82	1.768
Adrenal_gland	145	21	70	51	51	81	64	1.266
Artery_aorta	224	21	69	54	51	138	86	1.605
Artery_coronary	133	21	69	54	52	77	56	1.375
Artery_tibial	332	20	70	53	51	213	119	1.79
Brain_amygdala	72	20	70	60	58	50	22	2.273
Brain_anterior_cingulate_cortex_(BA24)	84	20	70	60	58	61	23	2.652
Brain_caudate_(basal_ganglia)	117	20	70	60	58	85	32	2.656
Brain_cerebellar_hemisphere	105	20	70	59	56	74	31	2.387
Brain_cerebellum	125	20	70	59	57	84	41	2.049
Brain_cortex	114	20	70	59	57	77	37	2.081
Brain_frontal_cortex_(BA9)	108	23	70	60	58	77	31	2.484
Brain_hippocampus	94	20	70	60	57	65	29	2.241
Brain_hypothalamus	96	20	70	60	58	71	25	2.84
Brain_nucleus_accumbens_(basal_ganglia)	113	20	70	60	57	79	34	2.324
Brain_putamen_(basal_ganglia)	97	20	70	59	57	69	28	2.464
Brain_spinal_cord_(cervical_c-1)	71	22	70	59	57	43	28	1.536
Breast_mammary_tissue	214	21	70	53	51	124	90	1.378
Cells_EBV-transformed_lymphocytes	118	21	70	50	48	75	43	1.744
Cells_transformed_fibroblasts	284	21	70	53.5	51	181	103	1.757
Colon_sigmoid	149	21	70	56	54	88	61	1.443
Colon_transverse	196	21	70	50	48	115	81	1.42
Esophagus_gastroesophageal_junction	153	21	70	53	51	94	59	1.593
Esophagus_mucosa	286	21	70	52.5	50	179	107	1.673
Esophagus_muscularis	247	21	70	50	49	157	90	1.744
Heart_atrial_appendage	194	20	70	55	54	126	68	1.853
Heart_left_ventricle	218	20	70	53	51	142	76	1.868
Liver	119	21	69	55	54	78	41	1.902
Lung	320	21	70	54	52	213	107	1.991
Muscle_skeletal	430	20	70	54.5	52	274	156	1.756
nerve_tibial	304	20	70	54	52	199	105	1.895
Ovary	97	21	69	51	50	97	NA	NA
Pancreas	171	21	70	51	50	102	69	1.478
Pituitary	103	20	70	59	57	74	29	2.552
Prostate	106	21	70	50.5	49	106	NA	NA
Skin_not_sun_exposed_(suprapubic)	250	20	70	55	53	164	86	1.907
Skin_sun_exposed_(lower_leg)	357	21	70	55	52	226	131	1.725
Small_intestine_terminal_ileum	88	21	70	49.5	48	51	37	1.378
Spleen	104	21	68	50	48	60	44	1.364
Stomach	193	21	70	51	48	111	82	1.354
Testis	172	21	70	52	50	172	NA	NA
Thyroid	323	20	70	55	53	211	112	1.884
Uterus	83	21	69	50	48	83	NA	NA
Vagina	96	21	69	51	50	96	NA	NA
Whole_blood	393	20	70	54	52	249	144	1.729

### Age Prediction Based on Single Tissue

**Figure 1:**
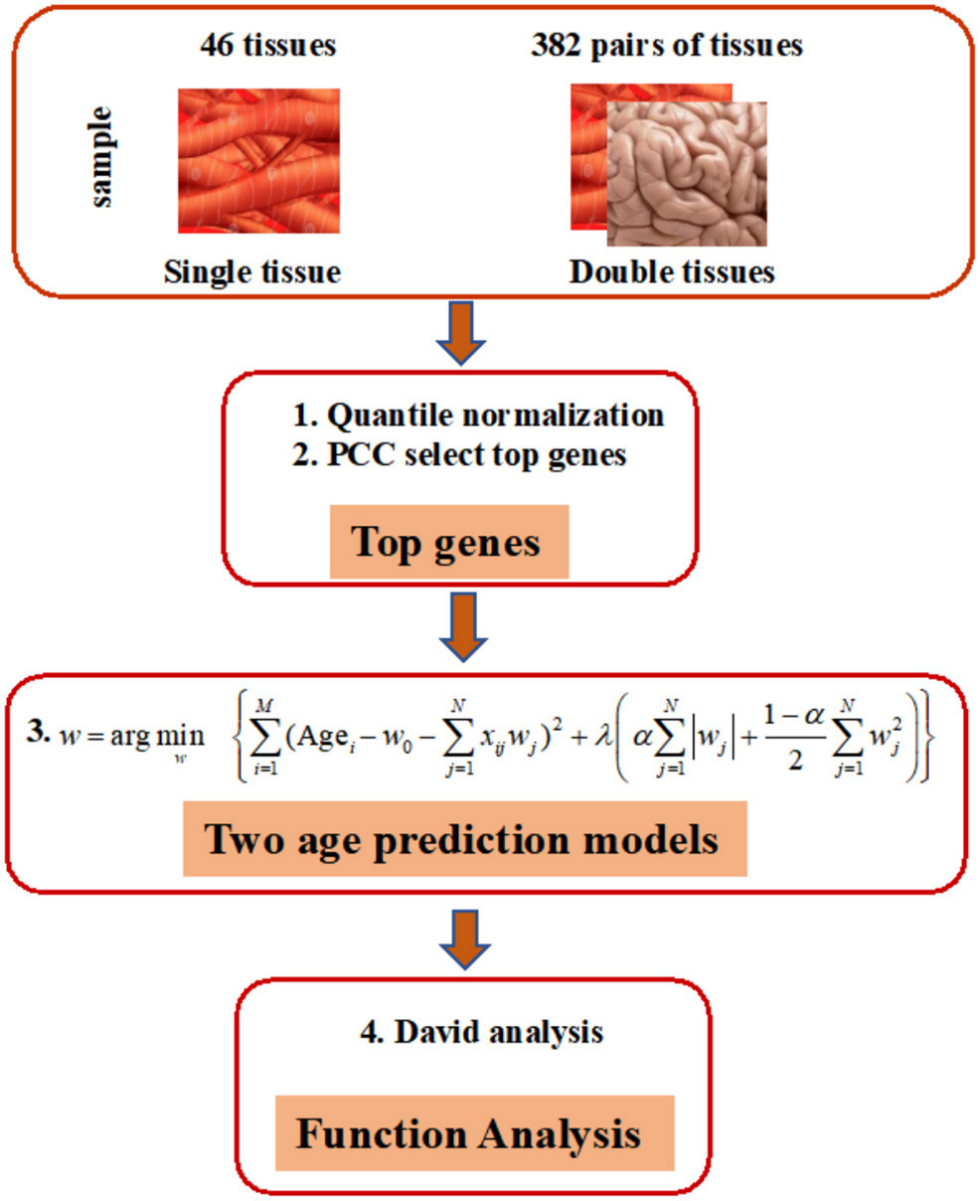
Overview of elastic net method for building age-prediction model. 1. Normalize the original gene expression data from GTEx via quantile normalization. 2. Select the top 50, 100, 200, 400, 600, 800, 1,600, 3,200, and 6,400 genes, obtained via the Pearson correlation of the age and corresponding gene expression, and build the age-prediction model for each of 46 tissues. 3. Construct age-prediction model for multiple tissues as was done for single tissues. Because overlapping samples among three tissues are often less than 70, only two-tissue studies are contained in the current study. 4. Use the selected genes for DAVID analysis.

As shown in Figure 1, our prediction framework has multiple steps. First, we rank the genes in each tissue based on the PCC of donor age and the corresponding gene expression. Top age-associated genes in one single or two tissues were then used to construct features in an elastic net regularization model, which is a sparse learning model capable of handling data with small sample sizes but numerous features (Zhou and Hastile, 2005). The parameters of the models were tuned through 10-fold CV according to the RMSE. Functions of genes were annotated by the DAVID Tools (see “Methods” for detailed information).

**Figure 2:**
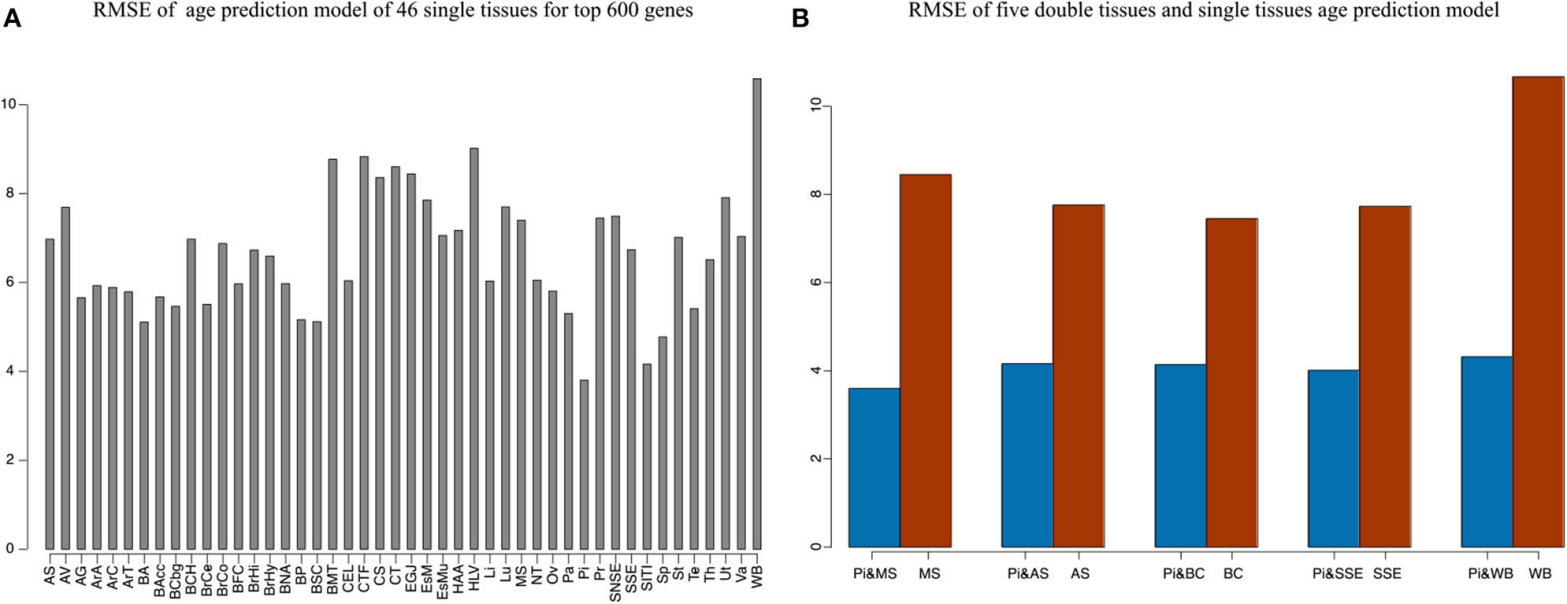
The accuracy of 46 single tissues and five double tissues in age prediction. **(A)** The RMSE of single tissue age predictors for the top 600 genes. We select the top 50, 100, 200, 400, 600, 800, 1,600, 3,200, and 6,400 genes, which are obtained via Pearson correlation of age and gene expression, and then build the age-prediction model across the 46 single tissues. Because the best predictive model appears in the top 600 genes, here we show the RMSE of the top 600 gene model. As can be seen from the figure, the minimum RMSE is 3.8, which corresponds to the age-prediction model of pituitary tissue. **(B)** Blue represents the RMSE of the top 600 genes of pituitary and the top 50 genes of muscle, adipose subcutaneous, brain cerebellum, skin sun exposed, and whole blood, and brown represents RMSE of the first 50 genes of muscle, adipose subcutaneous, brain cerebellum, skin sun exposed, and whole blood.

Our method was first applied to 46 single tissues, respectively. The performance of each tissue is listed in Table 2. As mentioned above, the number of top age-associated genes was taken as a parameter to our model. We selected the top 50, 100, 200, 400, 600, 800, 1,600, 3,200, and 6,400 genes and tested their performances by the 10-fold CV. It turns out that the number of top genes has some influence on prediction accuracy. The lowest RMSE (i.e., 3.8 years) was achieved for pituitary while selecting 600 genes. Pituitary is one of the most studied tissues and is highly associated with human aging (Seeman and Robbins, 1994). Other good tissues for age prediction include small intestine terminal ileum, spleen and testis, and brain/spinal cord. The most accessible tissue, whole blood, seems to be unsuitable for this task. Hannum et al. (2013) applied a blood gene expression profile to predict age based on a much larger sample size (488 in total). However, the RMSE is 7.22 years, which is comparable to our result. We also plotted the RMSEs for all other tissues (using the top 600 genes) in Figure 2A for a better view.

**Table 2:** Prediction accuracy by using single tissue.

**Tissue**	**Validation RMSE**								
	**50**	**100**	**200**	**400**	**600**	**800**	**1,600**	**3,200**	**6,400**
Adipose_subcutaneous	7.76	7.35	7.28	7.17	6.97	7.03	6.97	7.05	7.2
Adipose_visceral_(omentum)	8.49	8.35	8.02	7.86	7.69	7.78	7.67	7.95	7.6
Adrenal_gland	7.82	7.3	6.97	6.06	5.66	5.46	5.25	5.38	5.53
Artery_aorta	6.84	6.68	6.43	6.14	5.93	5.98	5.77	5.76	5.9
Artery_coronary	8.28	8.02	7.32	7	5.89	6.12	5.78	5.84	6.06
Artery_tibial	7.44	6.41	6.09	5.99	5.79	5.88	5.71	5.81	6.07
Brain_amygdala	7.11	6.52	6.31	5.62	5.11	5.27	5.23	5.41	5.39
Brain_anterior_cingulate_cortex_(BA24)	6.3	5.89	6.5	5.82	5.68	6	6.16	6.32	6.51
Brain_caudate_(basal_ganglia)	6.64	6.62	6.26	5.61	5.46	5.63	5.07	4.65	4.65
Brain_cerebellar_hemisphere	7.23	7.53	7.46	7.52	6.97	6.9	6.52	6.09	6.14
Brain_cerebellum	7.13	6.73	6.21	5.82	5.51	5.25	5.01	4.69	4.63
Brain_cortex	7.45	6.98	7.47	6.57	6.87	6.81	5.81	5.92	5.67
Brain_frontal_cortex_(BA9)	7.2	7.39	6.56	6.25	5.97	5.9	5.9	5.32	5.34
Brain_hippocampus	8.04	8.08	8.21	6.77	6.73	6.87	6.9	6.41	5.54
Brain_hypothalamus	6.91	7.05	6.91	6.59	6.6	6.43	6.29	6.19	6.59
Brain_nucleus_accumbens_(basal_ganglia)	7.22	6.56	6.15	6.53	5.98	5.51	5.73	5.33	5.43
Brain_putamen_(basal_ganglia)	7.22	7.09	6.3	5.56	5.16	5.19	5.55	5.52	5.8
Brain_spinal_cord_(cervical_c-1)	6.9	6.86	5.26	5.32	5.12	4.91	4.83	5	5.51
Breast_mammary_tissue	10.38	10	9.5	9.06	8.77	7.98	6.86	6.28	6.4
Cells_EBV-transformed_lymphocytes	8.86	8.18	7.56	6.29	6.04	5.68	5.64	5.87	6.78
Cells_transformed_fibroblasts	10.38	9.91	9.14	9.25	8.83	8.74	8.26	7.76	7.74
Colon_sigmoid	9.42	8.96	8.8	8.9	8.36	8.25	8.36	7.14	7.5
Colon_transverse	9.58	9.37	9.04	8.83	8.6	8.6	8.42	8.37	7.98
Esophagus_gastroesophageal_junction	8.94	9	8.91	8.61	8.44	8.35	7.56	7.18	6.86
Esophagus_mucosa	8.49	8.37	8.28	7.95	7.85	7.58	7.56	7.69	7.58
Esophagus_muscularis	7.78	7.65	7.81	7.69	7.06	6.91	6.55	6.04	6.38
Heart_atrial_appendage	8.66	8.57	7.55	7.44	7.17	7.12	6.65	5.93	5.96
Heart_left_ventricle	9.4	9.15	9.5	9.15	9.02	8.91	8.06	7.25	6.87
Liver	7.49	6.76	6.13	5.92	6.03	5.69	5.48	5.77	6.08
Lung	8.71	8.46	8.59	8.13	7.7	7.7	7.69	6.92	7.12
Muscle_skeletal	8.45	7.83	7.43	7.28	7.4	7.52	7.37	6.96	6.86
Nerve_tibial	6.81	6.54	6.19	5.88	6.05	6.22	5.96	5.71	5.74
Ovary	6.09	6.14	5.89	5.78	5.81	5.46	5.39	5.22	5.41
Pancreas	5.85	5.97	5.63	5.15	5.3	4.93	4.27	4.51	5.06
Pituitary	5.53	5.11	4.57	4.23	3.8	3.98	3.92	4.11	4.55
Prostate	8.86	8.91	8.68	8.04	7.45	7.4	6.88	6.87	6.57
Skin_not_sun_exposed_(suprapubic)	9.04	8.58	8.24	8	7.49	7.35	7.24	6.19	6.24
Skin_sun_exposed_(lower_leg)	7.73	7.35	7.11	6.79	6.74	6.8	6.52	6.25	6.11
Small_intestine_terminal_ileum	7.57	7.07	5.54	4.24	4.16	4.03	4.16	4.59	5.49
Spleen	6.83	6.16	6.22	5.18	4.77	4.52	4.71	5.1	5.3
Stomach	9.7	8.6	8.01	7.38	7.01	6.82	6.15	6.2	6.71
Testis	6.5	6.03	5.81	5.5	5.41	5.31	4.83	4.92	4.95
Thyroid	7.91	7.56	6.91	6.77	6.51	6.54	6.22	6.39	6.1
Uterus	6.64	6.86	7.59	7.67	7.91	7.76	7.53	7.24	7.23
Vagina	8.55	8.42	8.06	7.29	7.03	6.66	6.94	6.64	6.99
Whole_blood	10.67	10.6	10.68	10.53	10.58	10.48	10.19	10.03	10.08

*In this table the age-prediction model established with 46 tissues using the top 50, 100, 200, 400, 600, 800, 1,600, 3,200, and 6,400 genes with the highest age-related degree, respectively. Validation RMSE of 46 single tissues by 10-fold CV*.

### Age Prediction Using Multiple Tissues

**Figure 3:**
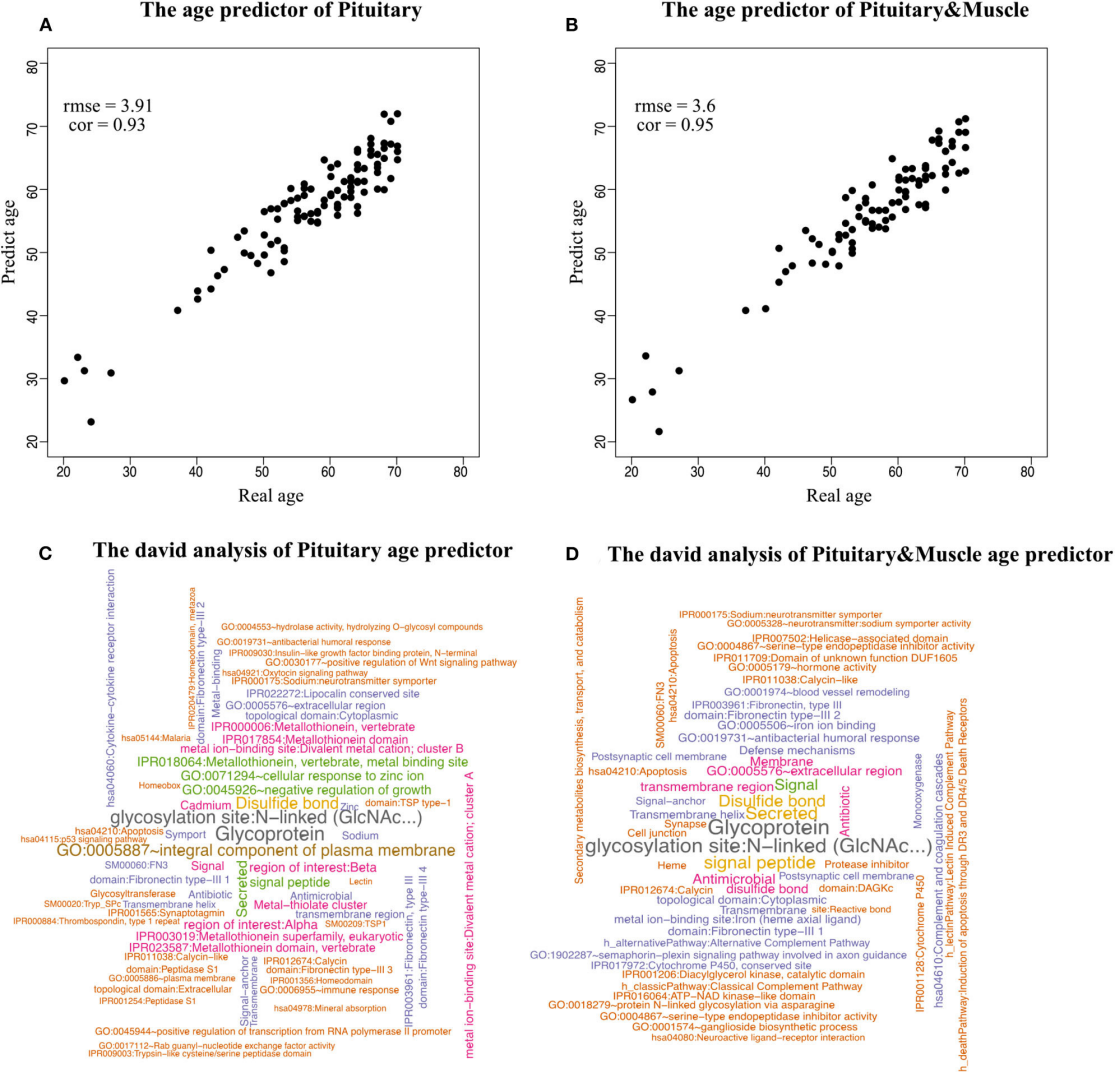
Scatterplot of age prediction and gene functional analysis. **(A)** Scatterplot of the pituitary age-prediction model for the top 600 genes in 46 single tissues. The RMSE is 3.8, and the PCC of real and predicted age is 0.93. **(B)** Scatterplot of pituitary for 600 genes and muscle skeletal for 50 genes age-prediction model. The RMSE is 3.6, and the PCC of real and predicted age is 0.95. **(C)** DAVID analysis of the age-prediction model in pituitary. **(D)** DAVID analysis of the age-prediction model in pituitary and muscle skeletal.

Because aging is a process associated with multiple tissues (Kujoth et al., 2005), it is reasonable to assume that combining multiple tissues can improve age-prediction accuracy. Because there are at least 71 samples in a single tissue, we selected people with at least 70 samples in two tissues for a relatively fair comparison, which derives 382 combinations in total. The combinations were used to train 382 elastic net models (Zhou and Hastile, 2005), whose performances were also evaluated by the 10-fold CV. The results show that it is possible to improve age prediction by combining two tissues. As we mentioned above, the best prediction RMSE for single tissue (3.8 years) was achieved at pituitary with 600 genes. We added 50, 100, 200, and 400 selected genes from one other tissue, including muscle skeletal, adipose subcutaneous, brain cerebellum, skin sun exposed, and whole blood, whose performances are listed in Table 3 and shown in Figure 2B. As can be seen, the validation RMSE decreases to 3.6 by combining 50 genes from muscle skeletal (see also Figures 3A,B). However, the prediction accuracy is worse when adding other tissues, indicating that different tissues might undergo aging at different rates or mechanisms. Generally speaking, the age-prediction accuracy is elevated with the increase of tissue number, which supports that aging is a concordant process involving multiple tissues (Kujoth et al., 2005).

**Table 3:** Prediction accuracy by combining double tissues.

**Tissues**	**Validation RMSE**			
	**600 + 50**	**600 + 100**	**600 + 200**	**600 + 400**
Pituitary&muscle skeletal	3.6	3.61	3.67	3.78
Pituitary&adipose subcutaneous	4.16	4.23	4.36	4.36
Pituitary&brain cerebellum	4.14	4.15	4.21	4.19
Pituitary&skin sun exposed	4.01	4	4.03	4.08
Pituitary&whole blood	4.32	4.31	4.45	4.64

*In this table a double age-predicting model composed of pituitary and muscle, adipose, brain, skin, and whole blood; 600 is the most age-related gene in pituitary and 50, 100, 200, and 400 are the most age-related gene in other five tissues. Validation RMSE of pituitary and five tissue models by 10-fold CV*.

### Effect of Model Parameters on Prediction Accuracy

In our model, we prefilter genes and only allow the top *N* genes as features to be selected by the elastic net model. There are two elastic net parameters, namely α, which controls the balance between lasso and ridge regression, and λ, the lasso parameter. Because the effects of α and λ have been extensively studied (Zhou and Hastile, 2005), we tested the effect of *N* on validation error in this study. For most prediction models with a small validation error, the number of genes involved in the model ranges from 300 to 1600. As an indication, only a small or moderate portion of genes are necessary to predict age. This finding is also supported by other studies (Bocklandt et al., 2011; Hannum et al., 2013), in which 200 methylation markers are used to predict the biological age of individuals. The parameters of the best model (e.g., “pituitary&muscle”) are α = 0, λ = 0.5, *w*_0_ = 49.1, that is, age = 49.1 − 0.5534609 × RF00019 + 0.4345046 × *RASSF8* + 0.4238481 × *ALOX15B* + …

The model has an intercept of 49.1 years, which is quite close to the mean age of the samples 50.81.

### Optimal Gene Set of Predicted Age and Functional Analysis

For the best prediction model, we listed the top 50 genes (according to the absolute value of coefficients) and their coefficients in Table 4. Among the top 50 genes, 49 are from pituitary, and only 1 is from muscle (ranked at 15). Interestingly, most of the top genes are age-associated. For example, *RASSF8* (ras association domain-containing protein 8), ranks second in the list. *RASSF8* encodes a protein that is a member of the transmembrane 4 superfamily and is a lung tumor–suppressor gene candidate. It plays important roles in the regulation of localization, methylation, cell–cell adhesion, cell migration, cell death, response to hypoxia, mitosis, cell growth, wound healing, contact inhibition, and epithelial cell migration (Falvella et al., 2006; Wang et al., 2017; Karthik et al., 2018; Shi L. et al., 2018). Accumulated evidence suggests that *RASSF8* is associated with aging (Geigl et al., 2004; Shi Z. et al., 2018; Pagliai et al., 2019). Similarly, *ALOX15B* (Arachidonate 15-Lipoxygenase Type B), which ranks third on the list, is a protein-coding gene. Diseases associated with *ALOX15B* include autosomal recessive congenital ichthyosis and prostate cancer (Bhatia et al., 2005; Ginsburg et al., 2016; GeneCards, 2020). This gene is a senescent gene, which can also affect human aging with its expression increasing when prostate epithelial cells become senescent (Bhatia et al., 2005; Alfardan et al., 2019). In addition to age-associated genes, there are also many genes whose association with aging is unknown. For example, no association with aging could be identified in the literature for the top gene *RF00019* on the list. In the future, further studies might be needed to elucidate the mechanism for age-dependent functions of *RF00019*.

**Table 4:** Best models for age prediction using pituitary & muscle skeletal tissue.

**Gene symbol**	**Coefficient**	**Tissue**	**Gene symbol**	**Coefficient**	**Tissue**
Intercept	49.1				
RF00019	−0.5534609	Pituitary	HMGN2P46	−0.265154	Pituitary
RASSF8	0.43450456	Pituitary	AIPL1	−0.262319	Pituitary
ALOX15B	0.42384809	Pituitary	AC079922.1	−0.2613869	Pituitary
IGSF1	−0.3815586	Pituitary	CYP3A5	0.25593725	Pituitary
MAOA	0.3779751	Pituitary	MIR3186	−0.248713	Pituitary
PIGP	−0.3643882	Pituitary	FA2H	−0.2478653	Pituitary
AC138904.1	−0.3590232	Pituitary	LZTS1	−0.2453074	Pituitary
ITGA10	0.34749327	Pituitary	FKBP5	−0.2403517	Pituitary
CYP51A1P2	−0.3468059	Pituitary	HTN3	0.23757784	Pituitary
FABP6	0.33526575	Pituitary	VNN3	0.23713188	Pituitary
AC007938.1	−0.3287363	Pituitary	MMP11	−0.2370928	Pituitary
LINC01315	−0.3252791	Pituitary	PADI2	0.23575174	Pituitary
AL596325.2	0.32297086	Pituitary	NANOGNBP3	0.23556292	Pituitary
LINC00662	0.3151238	Muscle	ST6GALNAC5	−0.2348075	Pituitary
CATSPERB	0.31335041	Pituitary	C7	−0.2308648	Pituitary
MUC1	0.31188538	Pituitary	KCNMB2-AS1	0.22953261	Pituitary
NBEAP3	0.29659649	Pituitary	DQX1	−0.2276446	Pituitary
SNAI3	−0.2943786	Pituitary	GSTM4	0.22188874	Pituitary
HIST1H1C	0.29287356	Pituitary	AC021016.1	0.22063205	Pituitary
LINC02232	0.28356117	Pituitary	FER1L4	0.2180329	Pituitary
S100A1	0.28252535	Pituitary	LY6G5B	0.21750613	Pituitary
KMO	0.27801131	Pituitary	ZBTB16	−0.2170829	Pituitary
HLA-DOB	0.27540573	Pituitary	FCF1P1	−0.2147114	Pituitary
AC124947.1	0.26677666	Pituitary	CHRNA1	0.21457823	Pituitary
KCNK4	−0.2667203	Pituitary	MGAT5	−0.2125122	Pituitary

*In this Table the coefficient of the pituitary and muscle combination model in Table 3. Here, we list the top 50 genes in the model. Coefficient indicates the weight of the gene in the age-prediction model*.

### Functional Annotation Clustering of Top Genes

To identify the biological processes associated with genes in the prediction model, we performed functional annotation analysis using the DAVID tools (Huang et al., 2009), a web-accessible set of tools that allow researchers to infer the biological meaning behind large lists of genes. Because our focus is on enriched functional categories rather than on individual genes, we selected the functional clustering with adjusted *P* < 0.05. The top cluster is related to glycoprotein (*P* = 1.79 × 10^−8^). Histidine-rich glycoprotein (HRG) is present at high levels in plasma, and it is synthesized by parenchymal liver cells and transported as a free protein as well as being stored in α-granules of platelets and released after thrombin stimulation (Blank and Shoenfeld, 2008). Levels of HRG variants in human blood are associated with chronological age and predict mortality (Hong et al., 2019). Also noteworthy were clusters related to age, for instance, GO:0045926~negative regulation of growth (*P* = 1.08 × 10^−4^) (Figures 3C,D).

## Discussion

Each human individual has two “ages.” One is the chronological age defined by the time that has passed since birth, and the other is biological age, which describes a shortfall between a population cohort average life expectancy and the perceived life expectancy of an individual of the same age (Jackson et al., 2003). An accurate estimation of biological age is helpful in studying aging, and several approaches have been proposed so far (Borkan and Norris, 1980; Dubina et al., 1983; Hannum et al., 2013). The aging prediction strategy in this study reflects the donor's biological age, effectively providing a possible way to identify key genetics or environmental factors that lead to younger biological age than the chronological age.

By constructing elastic net models, we can predict human age as well as identifying genes strongly associated with human aging. For example, *RASSF8* and *ALOX15B* have been studied to be associated with human aging and age-associated diseases. The function enrichment analysis revealed some common functions, such as glycoprotein and signal peptide in prediction models of multiple tissues, suggesting their general association with aging. In the future, we will identify tissue-common and tissue-specific aging genes and functions.

Our results suggest that the expression level of a small number of genes can reliably predict human age. In the single-tissue model, the predicted age showed a higher deviation from the true chronological age compared to predictions based on two tissues. This reveals that tissues within the same individual have heterogeneous aging rates. The tissue specificity of aging is reported by studies performed in model organisms (Herndon et al., 2002; Libina et al., 2003; Niedernhofer, 2008). On the other hand, aging is a concordant process involving multiple tissues. Different tissues have different potentials for revealing the chronological age of the host, jointly considering that multiple tissues can reduce the variation derived from a single tissue. For instance, our results indicate that blood is a poor choice for age prediction although it is one of the most accessible tissues. In both validation and test data sets, predicted age is more easily deviated from chorological age in blood compared with other tissues. The poor prediction performance of blood is also supported by the other study using the human whole blood transcriptome (Hannum et al., 2013), suggesting that the blood transcriptome fluctuates more due to its frequent interactions with other tissues and environmental factors through circulation (Benetos et al., 1993; Franklin et al., 1997).

Some improvements can be expected to increase the prediction accuracy. First, only two tissues were considered in this study due to sample size limitation. In the future, we may include more tissues. Second, we only use gene expression to predict age. Many other molecular biomarkers have also been reported successfully in predicting human age, for example, methylation (Hannum et al., 2013) and telomere length (Harley et al., 1990; Benetos et al., 2001). Last, there are many choices of machine learning technologies that can be adopted, for example, support vector machine (Cortes and Vapnik, 1995) and neural network (Mcculloch and Pitts, 1990). Combining multiple types of genomics data and data analysis methods will certainly facilitate the prediction efficiency greatly (Dobin et al., 2013).

## Conclusions

We have developed a computational framework to predict individual age through age-associated gene expression of single and two tissues. The predicted age is an indicator of biological age reflecting the life span and true functionality of a human body. Although gene expression from a single tissue could be used to estimate individual chronological age, the prediction accuracy is improved by properly combining those with other tissues. Different tissues provide different potential in predicting age, more reliable gene expression–based age markers are obtained in pituitary and skeletal muscle compared with blood.

## Data Availability Statement

All datasets generated for this study are included in the article/supplementary material.

## Author Contributions

ZT, LC, JY, and GT conceived, designed, and managed the study. FW and JY performed the experiments. HL, QLi, ZY, QLu, and GT provided computational support and technical assistance. All authors approved the final manuscript.

## Conflict of Interest

JY, HL, QLi, ZY, QLu, and GT were employed by the company Geneis Beijing Co., Ltd. The remaining authors declare that the research was conducted in the absence of any commercial or financial relationships that could be construed as a potential conflict of interest.

## Abbreviations

GTEx: The Genotype-Tissue Expression
RMSE: The root-mean-square error
PCC: The Pearson correlation coefficient
CV: Cross-validation.
